# Bitter Taste Receptor Agonist Denatonium Inhibits Stemness Characteristics in Hematopoietic Stem/Progenitor Cells

**DOI:** 10.1093/stmcls/sxad075

**Published:** 2023-10-05

**Authors:** Valentina Pensato, Maria Antonella Laginestra, Paolo Falvo, Stefania Orecchioni, Giovanna Talarico, Elena De Marchi, Samantha Bruno, Sara Mongiorgi, Giulia Mitola, Francesco Bertolini, Elena Adinolfi, Michele Cavo, Antonio Curti, Valentina Salvestrini

**Affiliations:** Department of Medical and Surgical Sciences (DIMEC), University of Bologna, Bologna, Italy; Institute of Hematology and Center for Hemato-Oncology Research, Department of Medicine and Surgery, University and Hospital of Perugia, Perugia, Italy; Laboratory of Experimental Oncology, IRCCS Istituto Ortopedico Rizzoli, Bologna, Italy; Laboratory of Hematology-Oncology, European Institute of Oncology IRCCS, Milan, Italy; Onco-Tech Lab, European Institute of Oncology IRCCS and Politecnico di Milano, Milan, Italy; Laboratory of Hematology-Oncology, European Institute of Oncology IRCCS, Milan, Italy; Onco-Tech Lab, European Institute of Oncology IRCCS and Politecnico di Milano, Milan, Italy; Laboratory of Hematology-Oncology, European Institute of Oncology IRCCS, Milan, Italy; Onco-Tech Lab, European Institute of Oncology IRCCS and Politecnico di Milano, Milan, Italy; Department of Medical Sciences, Section of Experimental Medicine, University of Ferrara, Ferrara, Italy; Department of Medical and Surgical Sciences (DIMEC), University of Bologna, Bologna, Italy; Department of Biomedical and Neuromotor Sciences - DIBINEM, University of Bologna, Bologna, Italy; Laboratory of Hematology-Oncology, European Institute of Oncology IRCCS, Milan, Italy; Onco-Tech Lab, European Institute of Oncology IRCCS and Politecnico di Milano, Milan, Italy; Laboratory of Hematology-Oncology, European Institute of Oncology IRCCS, Milan, Italy; Onco-Tech Lab, European Institute of Oncology IRCCS and Politecnico di Milano, Milan, Italy; Department of Medical Sciences, Section of Experimental Medicine, University of Ferrara, Ferrara, Italy; Department of Medical and Surgical Sciences (DIMEC), University of Bologna, Bologna, Italy; IRCCS Azienda Ospedaliero-Universitaria di Bologna, Istituto di Ematologia “Seràgnoli” Bologna, Italy; IRCCS Azienda Ospedaliero-Universitaria di Bologna, Istituto di Ematologia “Seràgnoli” Bologna, Italy; IRCCS Azienda Ospedaliero-Universitaria di Bologna, Istituto di Ematologia “Seràgnoli” Bologna, Italy

**Keywords:** hematopoietic stem cells, bitter taste receptors, denatonium benzoate, bone marrow microenvironment, bitter compounds

## Abstract

Bone marrow microenvironmental stimuli profoundly impact hematopoietic stem cell fate and biology. As G protein-coupled receptors, the bitter taste receptors (TAS2Rs) are key in transmitting extracellular stimuli into an intracellular response, within the oral cavity but also in extraoral tissues. Their expression in the bone marrow (BM)-derived cells suggests their involvement in sensing the BM microenvironmental fluctuation.

In the present study, we demonstrated that umbilical cord blood (UCB)-derived CD34^+^ cells express fully functional TAS2Rs along with the signal transduction cascade components and their activation by the prototypical agonist, denatonium benzoate, significantly modulated genes involved in stemness maintenance and regulation of cell trafficking. The activation of these specific pathways was confirmed in functional in vitro experiments. Denatonium exposure exerted an antiproliferative effect on UCB-derived CD34^+^ cells, mainly affecting the most undifferentiated progenitor frequency. It also reduced their clonogenicity and repopulating potential in vitro. In addition, the TAS2R signaling activation impaired the UCB-derived CD34^+^ cell trafficking, mainly reducing the migration toward the chemoattractant agent CXCL12 and modulating the expression of the adhesion molecules CD62L, CD49d, and CD29.

In conclusion, our results in UCB-derived CD34^+^ cells expand the observation of TAS2R expression in the setting of BM-resident cells and shed light on the role of TAS2Rs in the extrinsic regulation of hematopoietic stem cell functions.

## Graphical Abstract

**Graphical Abstract:**
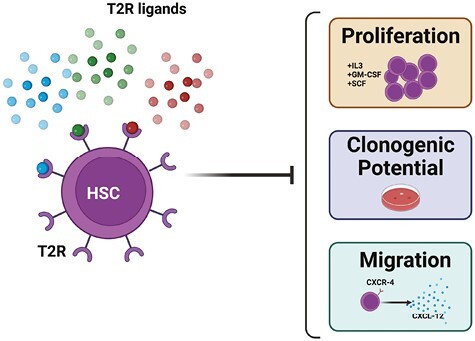

**Significance Statement**

Recent studies have highlighted an extra-oral expression of bitter taste receptors, TAS2Rs, questioning their exclusive role as sensors of bitter taste and expanding the spectrum of their putative functions. This work broadens the observation of TAS2R expression in the setting of BM-resident cells and sheds light on the TAS2R role in the extrinsic regulation of hematopoietic stem cell functions. Despite the mechanism and the involvement of specific TAS2Rs in each process remain to be clarified, our data suggest that a plethora of compounds, both endogenous and extrinsic, may interact with TAS2Rs affecting hematopoietic stem cell functions. Thus, it raises the possibility that off-target activation of TAS2Rs may play a role beyond normal physiology and mediate unexpected responses to, eg, drugs, many of which are bitter.

## Introduction

The bone marrow (BM) microenvironment provides complex and dynamic cues that play significant roles in controlling the cell fate direction.^1^ The cross-talk between hematopoietic stem/progenitor cells (HSPC) and their surrounding microenvironment is fundamental to a variety of processes, including proliferation, differentiation, and cell migration.^2^ Among receptors through which HSPCs respond to environmental fluctuation, most fall into the family of G protein-coupled receptors (GPCRs).^3^

Bitter taste receptors (TAS2Rs) belong to the GPCR Frizzled/Taste2 family and consist of 25 functional isoforms in humans.^4^ TAS2Rs were initially thought to be exclusively expressed in the oral cavity as a central warning system against the ingestion of dangerous and toxic substances. In the last 15 years, emerging evidence has shown that TAS2Rs and their downstream signaling targets are also expressed in many extra-oral tissues and have been involved in the physiology of the respiratory, digestive, endocrine, and genitourinary systems, as well as of the heart and the brain.^5-10^ Although their extra-oral function still needs to be deepened, their role in regulating physiological and pathophysiological processes^11^ is becoming increasingly evident, also in cancer settings.^12-17^

Of note, the expression of TAS2Rs has also been observed in cells that reside within the BM microenvironment, such as BM-derived mesenchymal stromal cells,^18^ osteoclast and osteoblast cells,^19^ along with blood cells.^20-24^ The bitter receptor TAS2R38 was proposed as a novel receptor on neutrophils for the quorum-sensing molecules produced by *Pseudomonas aeruginosa* and other Gram-negative bacteria^20,21^ and is expressed by resting and activated human lymphocytes, suggesting a role for this receptor family in the pathogen response of the adaptive immune system.^24^ TAS2R expression has also been demonstrated in circulating human monocytes, natural killer cells, B cells, and polymorphonuclear leukocytes.^25^ Their activation enhances phagocytosis in human monocyte-derived macrophages^22^ and inhibits the release of histamine and PGD2 from IgE-receptor-activated primary human mast cells.^23^ To date, there are no data about TAS2R expression in the HSPCs.

In the present study, we provided a comprehensive characterization of TAS2R expression in the HSPC compartment, and we studied the effect of HSPC exposure to denatonium benzoate (DEN), a widely used bitter taste agonist which has been demonstrated to activate TAS2Rs on various cell types.^26-30^ Our results showed that DEN affects the HSPC compartment, suggesting a role for bitter compounds in modulating stem cell fate.

## Materials and Methods

### Umbilical Cord Blood-Derived CD34^+^ Cells Isolation and Culture

Umbilical cord blood (UCB) samples were obtained from the Struttura Semplice Banca dei Tessuti del Sangue Cordonale e Biobanca, SIMIT A.M. BO. AOU of Bologna. Mononuclear cells (MNCs) were isolated by a density gradient centrifugation (Lympholyte, Cederlane). The enrichment of CD34^+^ cells from UCB-derived MNCs was obtained using an immunomagnetic separation according to the manufacturer’s instruction (MiltenyiBiotec). UCB-derived CD34^+^ cells were cultured in Dulbecco’s Modified Eagle Medium (DMEM) (Sigma Aldrich), supplemented with 10% heat-inactivated fetal bovine serum (FBS, Gibco), 1% Penicillin/Streptomycin (Pen/Strep) (MP Biomedicals), 1% Hepes buffer (Corning), SCF (50 ng/ml), IL-3 (50 ng/ml), GM-CSF (10 ng/ml) (MiltenyiBiotec) and maintained at 37°C in a humidified 5% CO_2_ incubator, with or without increasing doses of DEN (#D5765-1G), quinine (#145904-10G) or chloroquine (#C6628) (all from Sigma Aldrich). The Ethics Committee of Policlinico S. Orsola-Malpighi, University Hospital of Bologna, approved this research (approval code: 94/2016/O/Tess).

### In Silico Analysis

To evaluate the TAS2Rs expression level in UCB-derived CD34^+^ cells, we used raw data from 12 different single human donors from the publicly available gene expression profile dataset GSE19835.^31^ Background Correction and Normalization of Agilent-Whole Human Genome Microarray 4 × 44K were performed using the Agi4 × 44PreProcess package from R/Bioconductor, and the resulting normalized expression values were log_2_ transformed.

TAS2R gene expression levels were also performed in normal hematopoietic stem and progenitor cells isolated from 7 BM healthy donors from a published gene expression profile dataset GSE63270.^31^ Raw data were normalized using Robust Multi-Array Average (RMA), and log_2_ was transformed using the Oligo package^32^ from R/Bioconductor version 3.6.1.

### RNA Extraction and qRT-PCR

Total RNA was isolated using a Rneasy Micro kit (Qiagen) according to the manufacturer’s instructions and quantified by a Nanodrop ND-1000 spectrophotometer (Thermo Fisher Scientific). RNA samples were treated with DNase (Thermo Fisher Scientific) and reverse transcribed.^32^ The qRT-PCR reactions were performed using a 96-well Optical Reaction Plate and an ABI-PRISM 7900 Sequence Detection System (Thermo Fisher Scientific). The threshold cycle (C_t_) values for target genes and endogenous reference genes (Supplementary Table S1) were determined automatically. Relative quantification was calculated using the *Δ*Ct comparative method.^32^ cDNA from Universal RNA (Agilent genomics) was used as the reference sample. All reactions were performed in duplicate.

### Immunofluorescence

IF staining for TAS2Rs was performed on cytospins prepared from 1 × 10^6^ UCB-derived CD34^+^ cells. Cytospins were fixed with 4% paraformaldehyde for 10 minutes and permeabilized with PBS/Triton X-100 0.25% for 5 minutes, followed by 30 minutes blocking with PBS supplemented with 0.1% Tween and 1% BSA at room temperature (RT). The primary Abs (Supplementary Table S2) were incubated for 30 minutes at RT, and the corresponding secondary Abs (Supplementary Table S2) were incubated for 30 minutes at RT. Dapi antifade (Invitrogen) was finally added to identify the cell nucleus. Stained cells were examined under fluorescence Zeiss Imager. Z1 microscope and analyzed with ZEN3.1 Software.

### Viability Assay

5 × 10^4^ cells/100 µl culture medium were seeded into a 96-well microplate and treated as indicated. After culture, 20 µl CellTiter 96 AQueous One Solution reagent (Promega) was added to each well, and the microplate was incubated for 4 hours in standard conditions. The optical density value was measured by an ELISA plate reader (Multiskan Ex, Thermo Fisher Scientific) at a wavelength of 492 nm. Each condition was analyzed in triplicate.

### Cytosolic Ca^2+^ Concentration Measurement

Cytosolic-free Ca^2+^ concentration was measured in a thermostat-controlled (37°C) and magnetically stirred Cary Eclipse Fluorescence Spectrophotometer (Agilent Technologies) with the fluorescent indicator fura-2/AM as previously described.^17,33^ Briefly, 5 × 10^5^ cells were loaded with 2 µM fura-2/AM for 20 minutes in the presence of 1 mM CaCl_2_ and 250 µM sulfinpyrazone in the following saline solution: 125 mM NaCl, 5m M KCl, 1 mM MgSO4, 1 mM NaH_2_PO_4_, 20 mM HEPES, 5.5 mM glucose, 5 mM NaHCO_3_, pH 7.4. Subsequently, cells were rinsed and resuspended at a final concentration of 1 × 10^6^ cells/ml in the same buffer. Whenever required, cells were preincubated with 10 µM calcium chelator BAPTA-AM at 37°C for 30 minutes before fluorimetric measurements. Cells were stimulated with 10 mM DEN following signal stabilization. The excitation ratio and emission wavelengths were 340/380 and 505 nm, respectively.

### Gene Expression Profiling

GEP was performed on UCB-derived CD34^+^ cells cultured in the presence or absence of 0.5 mM DEN for 24 hours (*n* = 2 pools of 3 samples each) using human Clariom S Assay (Thermo Fisher Scientific) according to the manufacturer’s recommendations.

CEL files’ raw data were normalized using Robust Multi-array Average normalization (RMA), log-transformed, and annotated by pd.clariom.s.human R package.^34^ Differentially expressed genes between DEN-treated and untreated UCB-derived CD34^+^ cells were computed by Linear expression models using the Limma R/bioconductor package.^35^ We considered statistically significant differentially expressed the genes with a *P*-value < .05 and absolute |FC| = 1.5 (absolute |log2FC|= 0.5).

The functional Gene Ontology (GO) enrichment analysis was performed with Thomson Reuter’s MetaCore software suite (Clarivate Analytics, Philadelphia, PA, USA). Volcano Plot and Heatmap visualization were performed using functions available within R environment (https://www.r-project.org/).

### Colony-Forming Unit Assays

UCB-derived CD34^+^ cells were cultured in methylcellulose supplemented with cytokines (StemMACS HSC-CFU lite with Epo, MACS MiltenyiBiotec) at 500 cells/mL in 35-mm Petri dishes in the presence of DEN. In dedicated experiments, colony-forming unit (CFU) assays were preceded by a 6-day liquid culture in the presence of SCF (50 ng/ml), IL-3 (50 ng/ml), and GM-CSF (10 ng/ml), with or without DEN. Cell cultures were maintained at 37°C in a fully humidified atmosphere with 5% CO_2_, and after 14 days, CFU-Cs were scored under an inverted microscope (AXIOVERT 40CFL; Zeiss)

### Long-Term Culture-Initiating Cell Assay

The long-term culture-initiating cell (LTC-IC) assay was performed according to the manufacturer’s instructions (STEMCELL Technologies). Briefly, 5 × 10^3^ highly purified UCB-derived CD34^+^ cells were cultured on irradiated murine stromal cells (M210-B4) in a 24-well plate in the presence or absence (indicated as “control”) of DEN and maintained at 37°C, 5% CO_2_ for up to 5 weeks. DEN was added to the culture every week. After 5 weeks, the cells were harvested, the clonogenic potential of cultured cells was assessed in CFU assays, and the number of LTC-IC was calculated.^36^

### Proliferation Assay

UCB-derived CD34^+^ were stained with 5 μM green fluorochrome carboxyl fluorescein diacetate succinimidyl ester (CFSE; BioLegend) for 4 minutes at RT in PBS, 0.1% BSA, followed by the addition of ice-cold DMEM with 10% FBS. Cells were washed 3 times in an ice-cold medium and maintained in culture for 6 days in DMEM with 10% FBS supplemented with SCF (50 ng/ml), IL-3 (50 ng/ml), and GM-CSF (10 ng/ml) (MiltenyiBiotec), in the presence of 0.5 mM DEN. At the end of the culture, cells were analyzed by flow cytometry (CytoFLEX V0-B5-R3, Beckman Coulter) and FCS Express 4 analysis software (De Novo Software).

### Cell Cycle

UCB-derived CD34^+^ cells were permeabilized with PBS-0.1% NP-40 at 4°C for 15 min and then labeled with PI/RNase Staining Buffer (BD) at room temperature for 15 min. The DNA content was assessed by CytoFlex (Beckman Coulter), and the results were analyzed by Kaluza Analysis Software (Beckman Coulter).

### Flow Cytometry

Hematopoietic progenitor subpopulation (hematopoietic stem cells [HSC], multipotent progenitor [MPP], lymphoid-primed multipotent progenitor [MLP], common myeloid progenitor [CMP], granulocyte-macrophage progenitor [GMP], and megakarocyte-erythroid progenitor [MEP]) were characterized as indicated in Supplementary Fig. S1.

For the surface staining, cells were incubated with conjugated antibodies (Supplementary Table S3) at room temperature for 20 minutes. The stained cells were analyzed with the CytoFLEX V0-B5-R3 cytometer (Beckman Coulter) and Kaluza Analysis Software (Beckman Coulter).

### Homing Assay

Experiments involving animals were approved by the Italian Ministry of Health and have been done in accordance with the applicable Italian laws (D.L.vo 26/14 and following amendments), the Institutional Animal Care and Use Committee, and the institutional guidelines at the European Institute of Oncology. NOD-scid IL2Rgamma^null^ mice were transplanted intravenously via the tail vein with 175 × 10^3^ UCB-derived CD34^+^ cells, previously exposed to 0.5 mM DEN for 24 hours, after sublethal irradiation. Two days post-transplantation, cells were isolated from BM and spleen and analyzed using the following antibodies with Navios cytometer (Beckman Coulter): anti-humanCD45-APC (clone J33), anti-humanCD34-PC7 (clone 581) (Beckman-Coulter), anti-mouse CD45-PE (clone 30-F11) (BD), and 7-aminoactinomycin D (7AAD) (Sigma-Aldrich).

### Migration Assay

Cell migration was tested using transwell assays (diameter 6.5 mm, pore size 5μm Corning Costar). Briefly, 100 µl DMEM 10% FBS, containing 1 × 10^5^ cells with or without 0.5 mM DEN, was added to the upper chamber, while 600 µl medium, with or without 150 ng/ml CXCL-12 (Meridian Life Science), was added to the bottom chamber. After 4-hour incubation at 37°C in a 5% humidified CO_2_ atmosphere, inserts (upper chambers) were removed, and cells transmigrated into the lower chamber were recovered and counted. The number of migrating cells was measured with an inverted microscope (Nikon) using a 5-times magnification. In some experiments, UCB-derived CD34^+^ cells were preincubated for 24 hours with 0.5 mM DEN, which was washed out before the migration assay.

### Statistical Analysis

GraphPad Prism 7 software was used to perform statistical analyses, and the tests used are specified in the legend of each figure. Results are shown as mean ± SD of at least 3 separate experiments.

*P*-values <.05 were considered statistically significant and indicated as *P < .05, **P < .01, ***P < .001, and ****P < .0001.

## Results

### UCB-Derived CD34^+^ Cells Express TAS2Rs

**Figure 1.**
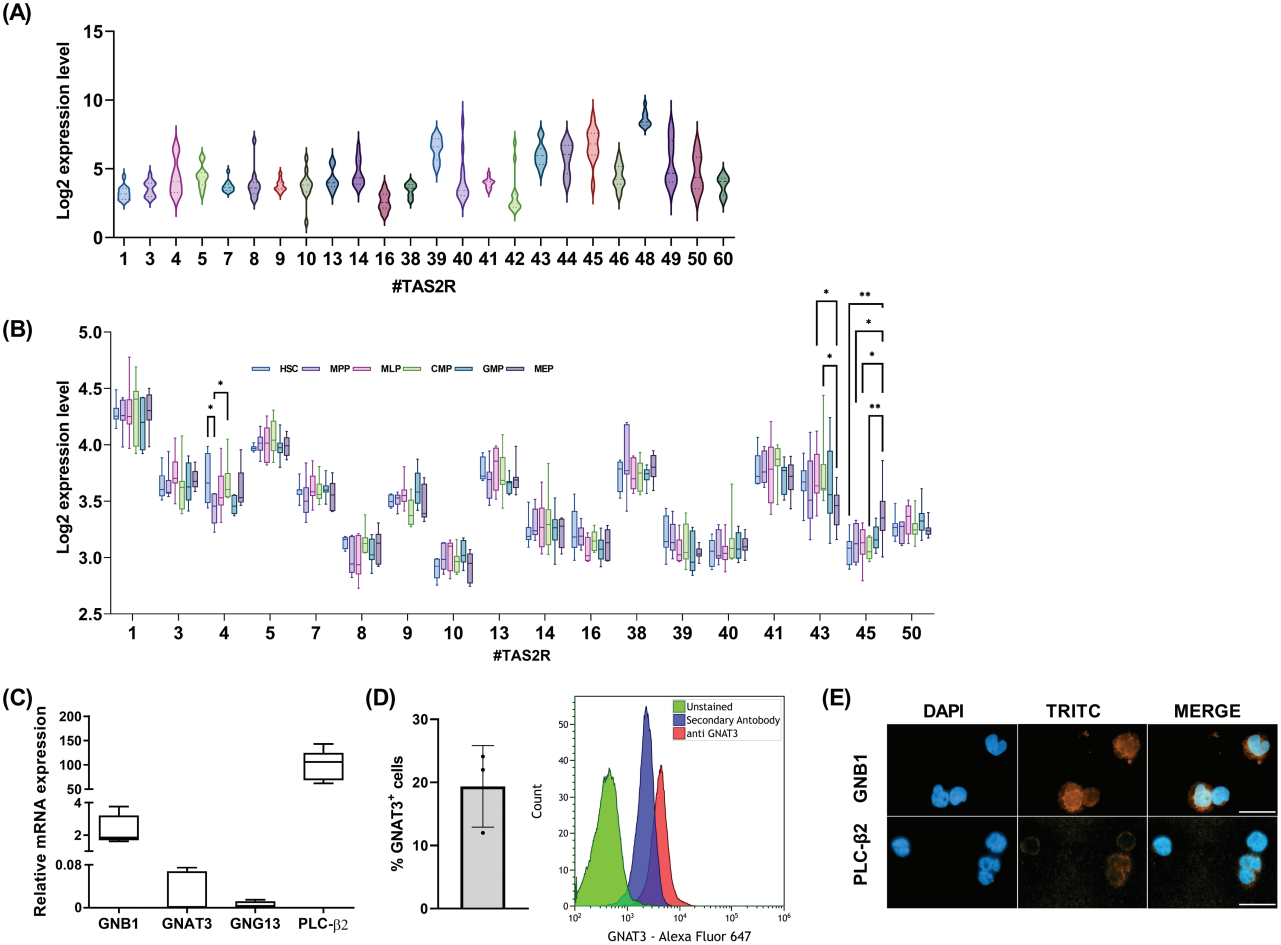
TAS2R expression in hematopoietic stem cells. TAS2R expression level distribution in (**A**) UCB-derived CD34^+^ from 12 CB samples (Whiskers min to max) (accession number: GSE19835) and in (**B**) hematopoietic stem cell (HSC), multipotent progenitor (MPP), lymphoid-primed multipotent progenitor (MLP), common myeloid progenitor (CMP), granulocyte-macrophage progenitor (GMP), and megakaryocyte-erythroid progenitor (MEP) from 7 BM healthy donors (accession number: GSE63270) (*TAS2R4*: MPP vs. HSC *P = .031, MPP vs. CMP *P = .039; *TAS2R43*: MEPs vs. CMPs *P = .011; *TAS2R45*: MEPs vs. HSCs **P = .002, MEP vs. MPP *P = .049, and MEP vs. CMPs **P = .004; 2-way ANOVA, Tukey’s multiple comparisons test). (**C**) qRT-PCR analysis of TAS2R downstream targets GNB1, GNAT3, GNG13, and PLC-β2 (*n* = 10). Relative target expression levels were calculated as described in A. (**D**) FACS analysis of GNG13 expression (*n* = 3). (**E**) Immunofluorescence analysis of GNB1, GNAT3, and PLC-β2 expression in UCB-derived CD34^+^ cells. 40× magnification, scale bar = 20 µm. Data represent mean ± SD.

Based on the emerging role of TAS2Rs in the hematological field, we investigated their expression and potential involvement in regulating HSPC function. To assess the TAS2Rs transcript level, we analyzed gene expression microarray datasets of 12 samples of CD34^+^ cells isolated from umbilical cord blood using the publicly available gene expression profile dataset GSE19835. Based on the log2 expression level, UCB-derived CD34^+^ cells are characterized by a heterogenous TAS2R gene expression (Fig. 1A). In particular, *TAS2R39*, *TAS2R43*, *TAS2R44*, *TAS2R45*, and *TAS2R48* showed a higher level of TAS2R mRNAs whereas *TAS2R16*, *TAS2R38*, *TAS2R42* showed a lower TAS2R transcript expression (Fig. 1A). We also performed TAS2R gene expression analysis in hematopoietic stem and progenitor cells isolated from 7 BM healthy donors, retrieved from GSE63270 (Fig. 1B). Despite the different TAS2R expression between stem cells from UCB and BM samples, HSCs and progenitor cells from BM expressed all TAS2R genes analyzed, suggesting that TAS2Rs are expressed by HSPCs regardless of the source. In addition, we observed some significant differences in TAS2R expression levels among progenitors. In particular, *TAS2R4* expression was decreased in MPP compared to HSCs and CMPs, *TAS2R43* expression was reduced in MEPs compared to CMPs and MLPs, and *TAS2R45* expression was increased in MEPs compared to HSCs, MPPs, MLPs, and CMPs (Fig. 1B).

As GPCRs, TAS2Rs work together with gustducins, a class of taste receptor-specific G proteins, activating the phospholipase C beta 2 (PLCβ2)/inositol-1,4,5-triphosphate (IP3) signaling pathway resulting in the calcium release from the endoplasmic reticulum.^35^ Thus, we found that freshly isolated UCB-derived CD34^+^ cells expressed the gustducins, GNB1, GNG13 and GNAT3, and the PLCβ2 (Fig. 1C-1E), indicating the necessary factors for the canonical TAS2R signaling pathway are present.

These data demonstrate that hematopoietic stem and progenitor cells express TAS2Rs coupled with the canonical signaling components.

### Bitter Taste Agonists Activate TAS2Rs in UCB-Derived CD34^+^ Cells

**Figure 2.**
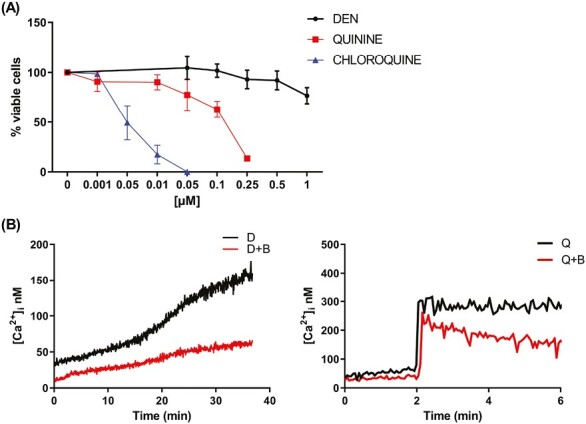
UCB-derived CD34^+^ cells express fully functional TAS2R receptors. (**A**) Cell viability detected by CellTiter 96 Aqueous One Solution assay in UCB-derived CD34^+^ cells treated for 48 hours with increasing doses of DEN (*n* = 7), quinine (*n* = 6), and chloroquine (*n* = 4). (**B**) Ca^2+^ release in UCB-derived CD34^+^ cells loaded with the Ca^2+^ indicator fura-2/AM and treated with 10 mM denatonium (indicated as D) or 50 µM quinine (indicated as Q) in the presence of BAPTA-AM (indicated as B) buffer. Data represent mean ± SD.

To evaluate the TAS2R functionality, we studied the effect of UCB-derived CD34^+^ cell exposure to 3 widely used bitter taste agonists^12,37-43^: DEN, which we have proved to affect leukemic cell functionality, quinine and chloroquine. First, we tested the agonists’ effective/not toxic dose by exposing UCB-derived CD34^+^ cells to increasing doses of the 3 compounds, based on each concentration range of T2R activation,^42^ and analyzed cell viability. After 48 hours of exposure, DEN did not affect UCB-derived CD34^+^ cell viability (Fig. 2A) or induce apoptosis (data not shown) at almost all the tested doses, except for 1 mM, which led to a 25% reduction of cell viability. In contrast, quinine reduced UCB-derived CD34^+^ cell viability in a dose-dependent manner, whereas chloroquine has been found to be toxic at all the tested doses (Fig. 2A). Then, we determined TAS2R activity by measuring the intracellular calcium rise following DEN and quinine administration. Increased calcium mobilization was observed in response to both DEN and quinine using the fura-2/acetoxymethyl ester (fura-2/AM) assay (Fig. 2B). The observed increase of intracellular calcium was due to store-operated calcium release, as expected from TAS2R activation, as demonstrated by its loss upon the addition of BAPTA-AM. Fig. 2B shows that BAPTA-AM, known to chelate calcium in intracellular stores, leads to a complete loss of DEN and quinine-dependent calcium increase into the cytosol, thus confirming TAS2R activation.

Together these data indicate that UCB-derived CD34^+^ cells express fully functional TAS2Rs, which are responsive to stimulation by agonists DEN and quinine.

### DEN Alters the Expression of Genes Involved in UCB-Derived CD34^+^ Cell Function

**Figure 3.**
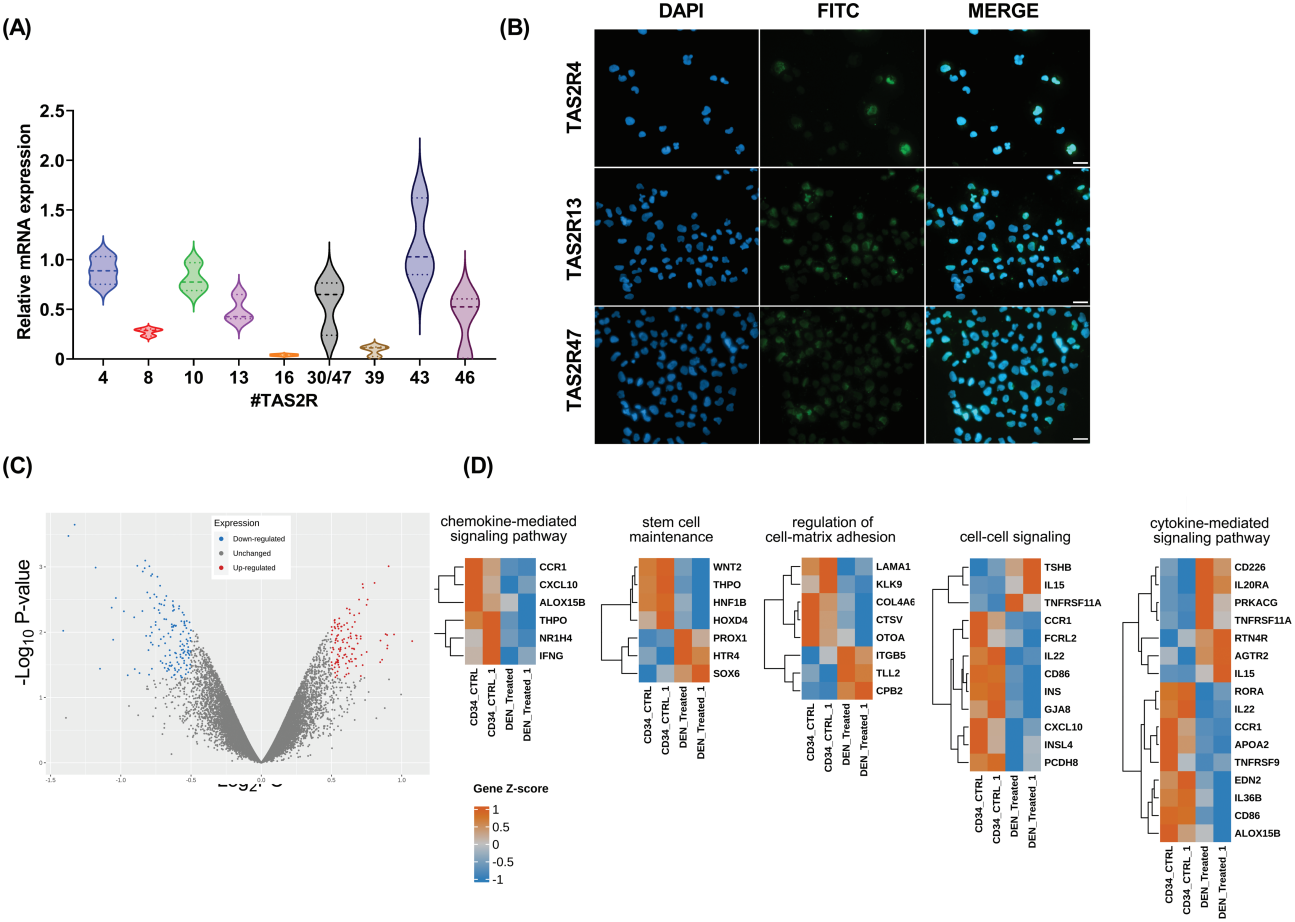
DEN exposure induces transcriptomic alterations in UCB-derived CD34^+^ cells. (**A**) DEN-activated TAS2R mRNA expression analysis by qRT-PCR in UCB-derived CD34^+^ cells (*n* = 11). Relative TAS2R expression levels were calculated using GAPDH as endogenous control, and the commercial cDNA pool as the reference sample was taken as 1 (2^−ΔΔCt^ method). (**B**) Immunofluorescence analysis of TAS2R4, TAS213, TAS2R30/47 expression. Nuclei were counterstained with DAPI (blue). 40× magnification, scale bar = 20 µm. (**C**) Volcano plot of differentially expressed genes identified between DEN-treated and untreated UCB-derived CD34^+^ cells. |log2-fold-change| threshold = 0.5, *P*-value threshold = .05. (**D**) Heatmap of differentially expressed genes enriched in chemokine-mediated signaling, stem cell maintenance, regulation of dell-matrix adhesion, cell-cell signaling, cytokine-mediated pathway, following 24-hour exposure to 0.5 mM DEN in UCB-derived CD34^+^ cells. The colour scale illustrates the relative expression level of a gene across all samples: orange represents an expression level above the mean and blue represents expression lower than the mean. *N* = 2 pools of 3 samples each.

To test the TAS2R function, we decided to use DEN as a model compound since, compared to quinine, it affected less UCB-derived CD34^+^ cell viability. DEN specifically activates 9 out of 25 TAS2Rs: TAS2R4, TAS2R8, TAS2R10, TAS2R13, TAS2R16, TAS2R30 (also known as TAS2R47), TAS2R39, TAS2R43, and TAS2R46. Therefore, we assessed their mRNA expression levels by qRT-PCR in an independent cohort of 11 samples of UCB-derived CD34^+^ cells. As shown in Fig. 3A, the results indicated that UCB-derived CD34^+^ cells express all DEN-related TAS2Rs. To confirm the expression of at least one T2R activated by DEN, we tested a selection of T2Rs at the protein level, including T2R4, T2R10, T2R13, and T2R30/47. Our analysis confirmed the protein expression of T2R4, T2R13, and T2R30/47 but not TAS2R10 in UCB-derived CD34^+^ cells (Fig. 3B). To assess changes in gene expression in response to DEN exposition in UCB-derived CD34^+^ cells, we performed GEP analysis after in vitro activation of the TAS2R pathway by DEN. Overall, the analysis identified 285 differentially expressed genes. In particular, DEN induced the upregulation of 126 genes and downregulation of 159 genes (Fig. 3C, Supplementary Table S4). To identify functional signatures associated with gene expression changes in response to DEN treatment, we performed GO enrichment analysis. Among several processes selected based on FDR ≤ 0.05, we found genes involved in stem cell maintenance (GO:2000036), regulation of cell-matrix adhesion (GO:0001952), cell-cell signaling (GO:0007267), chemokine-mediated signaling pathway (GO:0070098), cytokine-mediated signaling pathway (GO:0019221), (Fig. 3D, Supplementary Table S5). Notably, among the differentially expressed genes, we highlighted that DEN-treated cells were characterized by a reduced expression of genes involved in the induction of proliferation and differentiation, such as *IFNG*, *TPHO*, *CCR1*,^44^*TNFRSF9*,^45^*HOXD4*,^46^*CD86*,^47^ besides genes involved in the regulation of self-renewal and quiescence, such as *WNT2*,^48^*HOXC8*, or *PROX1*,^49^*ZFHX3*,^50^*AGTR2*, which were upregulated instead. Furthermore, DEN exposure altered the expression of genes involved in cell migration, adhesion to the extracellular matrix, and metalloproteinases, eg, *ITGB5*, *ITIH2*, *TLL2*, *C9*,^51^*ADGRD1*, which were upregulated, and *COL4A6*, *LAMA1*, *CEMIP*, *ADAMTS12*, which were downregulated, suggesting a role for TAS2R pathway in the regulation of UCB-derived CD34^+^ trafficking.

These findings suggest that DEN exerts a modulatory effect on the GEP of UCB-derived CD34^+^, mainly affecting the expression of genes that regulate stemness and cell trafficking.

### DEN Modulates UCB-Derived CD34^+^ Repopulating Potential and Proliferation In Vitro

**Figure 4.**
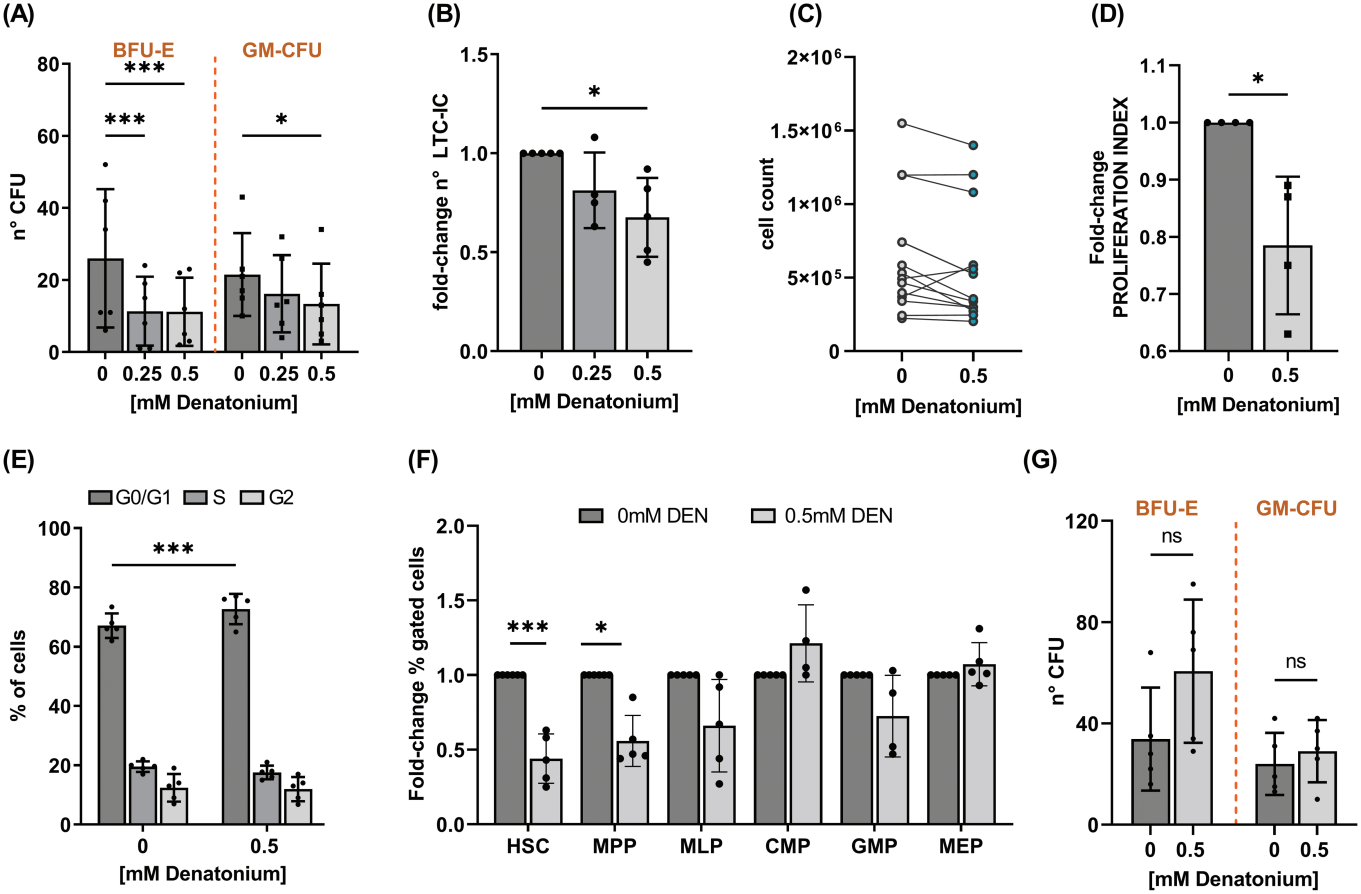
DEN modulates UCB-derived CD34^+^ proliferation and clonogenic efficiency. (**A**) The bar graphs indicate the number of CFU obtained from UCB-derived CD34^+^ cells cultured in semisolid medium in the presence of cytokines and increasing concentration of DEN (*n* = 6 *P = .023; 2-way ANOVA Dunnett’s multiple comparisons test). (**B**) UCB-derived CD34^+^ cells were cultured on irradiated M210B4 cells in the presence or absence of DEN for 5 weeks and then tested for their clonogenic potential. The bar graph represents the LTC-IC output. Data are shown as a fold-change of the LTC-IC number after DEN exposure compared to the control condition set as 1. LTC-IC in the control condition was 129 ± 36 (*n* = 5, *P = 0.042; one-way ANOVA Dunnett’s multiple comparisons test). (**C**-**G**) UCB-derived CD34^+^ cells were cultured for 6 days in the presence of SCF. IL-3 and GM-CSF with or without 0.5 mM DEN. At the end of the culture, the following tests were performed: (**C**) the cell counting (*n* = 14, *P = .032 paired *t*-test); (**D**) proliferation assay determined by CFSE staining and normalized to time 0. (Mean ± SEM, *n* = 4, *P* = .037, paired *t*-test); (**E**) analysis of the cell cycle distribution (*n* = 5, ***P = .0004 2-way ANOVA, Bonferroni’s multiple comparisons test); (**F**) FACS analysis of hematopoietic stem cell (HSC), multipotent progenitor (MPP), lymphoid-primed multipotent progenitor (MLP), common myeloid progenitor (CMP), granulocyte-macrophage progenitor (GMP), and megakaryocyte-erythroid progenitor (MEP). Data are shown as a fold-change of the percentage of cells after DEN exposure compared to the untreated condition set as 1. The percentage in the control samples was: HSC 0.18 ± 0.07, MPP 1.74 ± 0.42, MLP 2.44 ± 0.75, CMP 3.69 ± 1.08, GMP 1.54 ± 0.46, MEP 56.15 ± 12.03; (*n* = 5, HSC ***P = .0003, MPP *P = .011; 2-way ANOVA, Bonferroni’s multiple comparisons test); (**G**) CFU assay after 14 days of culture in semisolid medium in the presence of cytokines, obtained from UCB-derived CD34^+^ cells pretreated 6 days with 0.5 mM DEN. (*n* = 5, ns = not significant, 2-way ANOVA Dunnett’s multiple comparisons test). Data represent mean ± SD.

To investigate whether DEN may influence the HSPC functions in vitro, we first analyzed the DEN effect on UCB-derived CD34^+^ clonogenic capacity using CFU assay. As shown in Fig. 4A, after 14 days of culture, the clonogenic potential of UCB-derived CD34^+^ cells was negatively affected by the presence of the agonist, and the total colony output was significantly reduced compared to the control. Then, we determined the ability of DEN to modulate the long-term repopulating potential of UCB-derived CD34^+^ cells by quantifying LTC-ICs, that measure the clonogenicity of primitive hematopoietic progenitors. UBC-derived CD34^+^ cells were cultured on fibroblast feeder layers in the presence of DEN for 5 weeks. At the end of the culture, DEN was removed, and the cells were seeded in methylcellulose. The LTC-IC quantification showed that DEN exposure reduced the LTC-IC output compared to control cells (Fig. 4B), suggesting a role for TAS2R pathway activation in reducing more primitive progenitor frequency and thus affecting long-term repopulating potential. We next sought to assess the DEN effect on UCB-derived CD34^+^ cell growth. UCB-derived CD34^+^ cells were cultured in the presence of SCF, IL-3, and GM-CSF, with or without DEN. After 6 days, we found that UCB-derived CD34^+^ cell count was reduced, and the proliferation was significantly inhibited in the presence of DEN, primarily due to a G0/G1-phase arrest of the cell cycle, as shown in Fig. 4C-4E and Supplementary Fig. S2A. By analyzing the impact of DEN exposure on the frequency of the progenitor subsets at the end of culture, we observed that the inhibition was mainly related to the multipotent stem cell compartment, particularly to HSCs and MPP (Fig. 4F). No differences were observed in the frequency of the mature cell populations in DEN-treated compared to untreated cells (data not shown), suggesting that the TAS2R pathway does not regulate the differentiation process. At the end of the liquid culture, UCB-derived CD34^+^ cells were also plated in methylcellulose and tested for clonogenic capacity without adding DEN. Interestingly, previous DEN exposure did not affect UCB-derived CD34^+^ clonogenicity compared to untreated cells (Fig. 4G)

Together these data suggest that DEN may promote a quiescent state of UCB-derived CD34^+^ cells and reduce their repopulating potential in vitro in a nonpermanent way.

### DEN Modulates UCB-Derived CD34^+^ Migration Toward CXCL12

**Figure 5.**
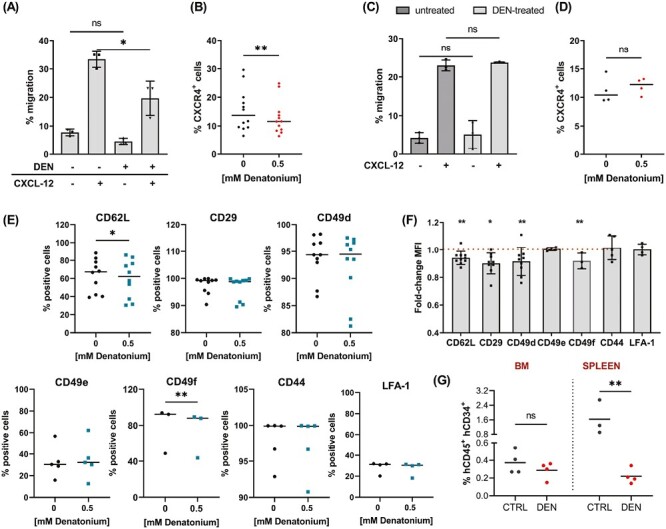
DEN modulates UCB-derived CD34^+^ migration capacity. (**A**) The bar graph shows the effect of the presence of 0.5 mM DEN in the upper chamber of the transwell system on the CXCL-12 induced chemotaxis (150 ng/mL) at 4 hours (*n* = 3, *P = .035, one-way ANOVA, Šidák’s multiple comparisons test) (**B**) FACS analysis of CXCR4 expression after 24 hours exposure to 0.5 mM DEN (*n* = 12, **P = .004, paired *t*-test). (**C**) The bar graph shows the effect of a 24 hours pretreatment with 0.5 mM DEN on the CXCL-12 induced chemotaxis (150 ng/mL) at 4 hours (*n* = 3, ns = not significant, one-way ANOVA, Šidák’s multiple comparisons test). (**D**) FACS analysis of CXCR4 expression 4-hour post a 24-hour pretreatment with 0.5 mM DEN (*n* = 4, ns = not significant, paired *t*-test). (**E**-**F**) FACS analysis of the adhesion molecules expression in UCB-derived CD34^+^ cells treated with 0.5 mM DEN (CD62L, CD49d, CD29 *n* = 10; CD49e, CD44, LFA-1 *n* = 5; CD49f *n* = 3; **P = .001, 2-way ANOVA, Šidák’s multiple comparisons test). The MFI in the control samples was: CD62L 7404 ± 2413, CD49d 13 395 ± 7459, CD29 6126 ± 2788, CD49e 9168 ± 1431, CD49f 2196 ± 111, CD44 158 083 ± 26 163, LFA1 1074 ± 48. (**G**) 175 × 10^3^ UCB-derived CD34^+^ cells were injected into sublethally irradiated NOD-scid IL2Rgamma^null^ immunodeficient mice after 24-hour incubation with or without 0.5 mM DEN. The human CD45^+^CD34^+^ cell percentage in the BM (ns = not significant, unpaired *t*-test) and spleen (*P = 0.027, unpaired *t*-test) of mice was evaluated by FACS analysis 24 hours after transplantation. Data represent mean ± SD.

GEP data showed the modulation of genes involved in regulating hematopoietic stem cell trafficking. Thus, we assessed the DEN effect on UCB-derived CD34^+^ migration ability. First, we evaluated the spontaneous migration in vitro using the transwell system. When increasing doses of DEN were added to the transwell upper chamber, we observed no differences in the percentage of migrated UCB-derived CD34^+^ cells after the direct exposure to DEN, indicating that the TAS2R signaling pathway does not interfere with the mechanism regulating the spontaneous migration (Supplementary Fig. S3A). Similar results were obtained even in the presence of a DEN gradient obtained by adding DEN to the transwell lower chamber (Supplementary Fig. S3B). Since the CXCL-12-CXCR4 axis is the key regulator of hematopoietic stem cell trafficking in the BM microenvironment, we wondered if DEN exposure affected the CXCL-12-CXCR4-dependent migration. DEN exposure significantly reduced UCB-derived CD34^+^ cell migration toward the chemoattractant agent CXCL-12 by decreasing the CXCR4 surface expression (Fig. 5A and 5B and Supplementary Fig. S3C). Nevertheless, if UCB-derived CD34^+^ cells were exposed to DEN for 24 hours and then tested for their migratory capacity toward a CXCL-12 gradient in the absence of DEN, they showed the same migration ability and CXCR4 expression as the control (Fig. 5C and 5D and Supplementary Fig. S3D), suggesting that DEN effects on CXCR4/CXCL12 axis are not permanent. Next, we investigated whether DEN affected UCB-derived CD34^+^ cell interaction with extracellular matrix components, which is pivotal in regulating cell motility. We treated UBC-derived CD34^+^ cells with DEN for 24 hours. Then, we analyzed the expression of the adhesion molecules L-selectin (CD62L), Integrin alpha 4 (CD49d) and Integrin beta 1 (CD29), Integrin alpha 5 (CD49e), Integrin alpha 6 (CD49f), CD44, and lymphocyte function-associated antigen 1 (LFA-1), which play a key role in the regulation of HSPC trafficking.^52^ As shown in Fig. 5E-5F and Supplementary Fig. S3E, DEN exposure induced a slight but significant reduction of the percentage of CD62L^+^ and CD49f^+^ cells and decreased the mean fluorescence intensity of CD62L, CD49d, CD49f, and CD29 on UBC-derived CD34^+^ cell surface, suggesting a reduced adhesion to endothelial cells and fibronectin after TAS2R pathway activation. In contrast, it did not affect CD49e, CD44, and LFA1 levels. Given that CD62L, CD49d, CD29, CD49f^+^, and CXCR4 are described as relevant for the homing of HSPC^52-54^ and TAS2R activation affects their expression, we considered whether DEN exposure affects UCB-derived CD34^+^ homing in vivo. To this end, UCB-derived CD34^+^ pretreated for 24 hours with DEN were transplanted in NSG mice and analyzed for their ability to migrate in the BM and spleen of the recipients. As shown in Fig. 5G, we found a trend, although not significant, between DEN exposure and a decrease in BM homing, and a significant reduction in migration toward the spleen.

Overall, these data suggest that DEN exposure may alter UBC-derived CD34^+^ cell trafficking mainly by interfering with the CXCR4-CXCL12 axis or modulating the adhesion to endothelial cells and extracellular matrix.

## Discussion

Initially described as a warning system to induce aversion toward noxious substances in the oral cavity, TAS2Rs have been recently shown to be expressed and functional in several extraoral tissues. As GPCRs, TAS2Rs are key in transmitting extracellular stimuli into an intracellular response, and their expression in the BM-derived cells^18-25^ suggests their involvement in sensing the BM microenvironmental stimuli. BM stimuli profoundly impact HSPC fate, and deepening the mechanism through which HSPCs respond to external fluctuation is essential to understanding HSPC biology. To date, there is no evidence of TAS2R expression on HSM.

The present study demonstrates that UBC-derived CD34^+^ cells express fully functional T2 receptors along with the signal transduction cascade components: α- and β-gustducins and PLC-β2.

Interestingly, the in silico analysis of gene expression microarray datasets revealed that HSCs and progenitor cells express the entire human TAS2R repertoire, regardless they are isolated from umbilical cord blood or BM, even though with a different expression profile, as expected for stem cells isolated from different sources.^55^ These observations, along with the demonstration of TAS2R expression in terminally differentiated blood cells,^20-25^ indicate that TAS2Rs represent a highly conserved receptor system during the differentiation process and, therefore, suggest that they are involved in regulating hematopoietic cell biology.

In recent years, increasing interest has been growing in the (patho-) physiological role of TAS2R in extraoral tissues; nevertheless, studying the TAS2R system and its downstream pathways remains still complex and challenging due to the impossibility of blocking all the 25 TAS2Rs using molecular techniques and the lack of specific antagonists. A shared strategy to analyze the TAS2R system is using TAS2R agonists. Hence, to assess the biological effect of TAS2R activation on UBC-derived CD34^+^ cells, we decided to use denatonium benzoate, a widely used bitter taste agonist,^30,37-41^ which we demonstrated to modify the transcriptomic profile and functions of acute myeloid leukemia cells.^17^ Our results demonstrate that UBC-derived CD34^+^ cells are responsive to TAS2R activation by DEN. Indeed, after TAS2Rs bind the ligand, gustducins initiate the dominant branch of the pathway, triggering PLC-β2-mediated inositol trisphosphate (IP3) production and intracellular calcium release from the endoplasmic reticulum.^35,56^ Even though we cannot exclude a concurrent TAS2R-independent DEN effect in UBC-derived CD34^+^ cells, ie, via direct interaction with ion channels,^34^ our data strongly suggest that TAS2R triggering is responsible for DEN-dependent intracellular calcium rise, supporting an on-target activity. Indeed, intracellular calcium chelation with BAPTA almost completely obliterated DEN-dependent calcium increase, thus demonstrating the presence of a store-operated release of the cation.

To assess the cellular processes modulated by TAS2Rs in UBC-derived CD34^+^ cells, we performed a GEP analysis after exposure to DEN. A significant number of genes were differentially expressed in UBC-derived CD34^+^ cells following DEN treatment. Interestingly, GO enrichment analysis indicated that relevant cellular processes, including stemness maintenance and regulation of cell trafficking, are targeted by TAS2R pathway induction. Our in vitro functional data corroborated the inhibitory effect of TAS2R activation by DEN on stemness characteristics of UCB-derived CD34^+^ cells. Indeed, DEN exposure reduced UCB-derived CD34^+^ cell proliferation capacity, in line with the observation of the antiproliferative effect of DEN in both healthy and cancer settings,^13,16,17,51,52^ and self-renewing potential. Interestingly, we observed that the inhibition was not permanent, suggesting that the TAS2R signaling required a constant activation to exert its inhibitory influence on stemness. Consistently with our data but in a cancer setting, Seo et al demonstrated the TAS2R involvement in the extrinsic regulation of stem cell functions.^15^ They showed that TAS2R over-expression in neuroblastoma cells is associated with the inhibition of their stemness characteristics; specifically, upregulation of endogenous TAS2R8 and TAS2R10 inhibited the expression of various cancer stem cell markers, including DLK1, CD133, Sox2, and Notch1, induces neuronal cell differentiation and inhibits self-renewal capacity.

In addition to repopulating potential, migration is another hallmark of HSPCs. Their ability to migrate from BM to blood and back to functional niches is responsible for maintaining the homeostasis of the hematopoietic system, and it is crucial in clinical settings such as BM transplantation.^57^ Interestingly, an increasing number of studies have demonstrated that migration capacity is affected by TAS2R signaling in various cell types. We demonstrated that TAS2R activation attenuates leukemia cell migration likely through inhibiting the CXCR4/CXCL-12 axis^17^; Sakakibara et al identified TAS2R38 as a migratory inhibitory receptor on the skin-infiltrating lymphocyte.^58^ Moreover, it has been reported that TAS2R4 and TAS2R14 activation inhibits chemotactic migration of metastatic breast adenocarcinoma cells, downregulating the matrix metalloproteinase (MMP)-9 secretion.^13^ Similarly, TAS2R8 and TAS2R10 overexpression in the BE(2)C neuroblastoma cell line inhibits cell migration and reduces the activity and expression of MMP-2.^59^ In line with these findings, our data showed that DEN exposure did not affect UCB-derived CD34^+^ cell spontaneous migration as well as the migration toward a DEN gradient but reduced UCB-derived CD34^+^ cell migration toward the chemoattractant agent CXCL-12 by decreasing the CXCR4 surface expression. The CXCR4/CXCL-12 axis is the key regulator of HSC trafficking in the BM microenvironment and is essential for the retention of HSPCs in the BM niche.^60^ Our data suggest that BM endogenous TAS2R ligands may affect HSPC fate, reducing their retention in the BM niche and encouraging their egress into the bloodstream. Supporting the hypothesis that TAS2R activation might promote HSPC egress into peripheral blood, a decrease in the expression of the adhesion molecules CD62L and VLA-4 (CD49d and CD29) has been observed after DEN exposure suggesting a reduced anchorage to the stroma and extracellular matrix of UCB-derived CD34^+^ cells. A decrease in the expression of the adhesion molecule and a concomitant reduction of CXCR4/CXCL-12-dependent migration could be mirrored in a decreased homing capacity.^61-63^ Our finding confirmed the inhibitory effect of DEN treatment on the homing ability of UCB-derived CD34^+^ cells, corroborating a role for TAS2R in regulating HSPC trafficking. Notably, we observed a more pronounced effect on homing toward secondary organs such as spleen. After transplantation in mice, primitive HSC (Sca1^+^ Lin^-^ cells) preferentially migrate to the bone marrow at any time, while the total Sca1^+^ cell population increases in the spleen by 20 hours.^64^ Drawing from this observation, our data suggest that DEN primarily influences the homing ability of the more differentiated progenitor cells.

The migratory capacity of the cells is important for hematopoietic recovery after stem cell transplantation,^63^ and it has been demonstrated that only CD34^+^ cells able to migrate to CXCL-12 have SCID-repopulating activity.^65^ In the light of our data, it is conceivable that TAS2R activation on HSPCs by endogenous or exogenous ligands could interfere with the hematologic recovery after cytotoxic treatments or stem cell transplantation, and thus, it should be taken into account in order to improve the efficacy of the therapeutic strategies.

Considering the wide expression of TAS2Rs in HSPCs, mature blood cells, bone cells, and generally, cells residing within BM, a question arises: what are the ligands for TAS2Rs present in the BM microenvironment? There are no proper studies about it, apart from the Lossow et al research identifying progesterone as a ligand for mouse TAS2R110 and TAS2R114.^66^ However, several ligands have been demonstrated to potentially activate TAS2R that may reach the BM and affect HSPC fate: first of all, therapeutic drugs, most of which are bitter tasting^67^; the bitter compounds in food, such as caffeine limonin or glucosinolates in crucifers,^42,68^ the postprandial concentration of which is increased in the bloodstream; endogenously produced factors, for example, amino acids hormones and vitamins^66,69,70^; but also bacterial products, such as quorum sensing molecules, that can interact with host GPCRs and induce innate immune responses.^30^ Although the mechanisms and the involvement of specific TAS2Rs in each process remain to be clarified, due to the high grade of redundancy in the expression of TAS2Rs that does not help identify a unique candidate liable for the TAS2R-mediated phenotype, our data suggest that a plethora of compounds, both endogenous and extrinsic, may interact with TAS2Rs expressed in HSPCs, affecting their fate and functions.

In conclusion, our results in UCB-derived CD34^+^ cells expand the observation of TAS2R expression in the setting of BM resident cells and shed light on the role of TAS2Rs in the extrinsic regulation of hematopoietic stem cell functions.

## Supplementary Material

sxad075_suppl_Supplementary_Material

## Data Availability

GEP data have been deposited in the Gene Expression Omnibus repository (GEO) and are accessible with the accession number GSE220210.
