# Supplementary material for: Bitter Taste Receptor Agonist Denatonium Inhibits Stemness Characteristics in Hematopoietic Stem/Progenitor Cells
Source: Stem Cells. 2023 Oct 5;42(1):42–54. doi: 10.1093/stmcls/sxad075 (PMC10787278; doi:10.1093/stmcls/sxad075)
Supplement: sxad075_suppl_Supplementary_Material [file sxad075_suppl_supplementary_material.pdf]

Supplementary Tables

Table S1. RT-PCR probes used in this study

Gene	accession number	Company	Probe ID	product size bp
TAS2R4	NM_016944	IDT	Hs.PT.58.24880205.g	131
TAS2R8	NM_023918	IDT	Hs.PT.58.27572746.g	133
TAS2R10	NM_023921	IDT	Hs.PT.58.25022966.g	112
TAS2R13	NM_023920.2	Applied Biosystem	Hs00256781_s1	132
TAS2R30/47	NM_001097643	IDT	Hs.PT.58.40967668.g	147
TAS2R39	NM_176881	IDT	Hs.PT.58.25428396.g	123
TAS2R43	NM_176884	IDT	Hs.PT.58.26835465.g	137
TAS2R46	NM_176887	IDT	Hs.PT.58.26491131.g	142
GAPDH	NM_002046	IDT	Hs.PT.58.40035104	123
GNAT3	NM_001102386.2	Applied Biosystem	Hs01385397_m1	109
GNB1	NM_001282538.1	Applied Biosystem	Hs00929799_m1	64
PLC-β2	NM_001284297.1	Applied Biosystem	Hs00190117_m1	69
GAPDH	NM_001289745.1	Applied Biosystem	Hs00266705_g1	74

Table S2. Primary and Secondary antibodies used in Immunofluorescence

Antibodies	Company	Catalog No.	Dilution
Goat anti-T2R4	Santa Cruz Biotechnology	sc-169494	1:50
Rabbit Anti-T2R13	ThermoFisher Scientific	PA5-39709	1:500
Goat Anti-T2R47	Santa Cruz Biotechnology	sc-34859	1:50
Mouse anti-PLC β2	Santa Cruz Biotechnology	sc-515912	1:25
Rabbit anti-GNB1	Abcam	ab137635	1:50
Swine anti-Rabbit secondary antibody	Dako	F0205	1:40
Rabbit anti-Goat secondary antibody	Dako	F0205	1:50
Goat anti-Mouse IgG (H+L) Highly Cross-Adsorbed Secondary Antibody, Alexa Fluor Plus 555	ThermoFisher	A32727	1:300
Rabbit anti-Goat IgG (H+L), Superclonal Recombinant Secondary Antibody, Alexa Fluor 555	ThermoFisher	A27017	1:400

Table S3. Antibodies used for FACS analysis

Antibodies	Company	Catalog No.
Mouse Anti-Human CD45RA PE-Cy™7	BD	337186
Mouse Anti-Human CD34 BV421	BD	562577
Mouse Anti-Human CD38 APC	BD	555462
Mouse Anti-Human CD90 FITC	BD	555595
Mouse Anti-Human CD123 PE	BD	340545
Mouse Anti-Human CD62L FITC	BD	555543
CD49d Mouse Anti-Human PE	BD	555503
Mouse Anti-Human CD29 APC	BD	303008
Anti-Human CD49e Antibody FITC	BioLegend	328007
Anti-Human CD11a/CD18 (LFA-1) APC/Fire™ 750	BioLegend	363419
Anti-human CD44 PerCP/Cyanine5.5	BioLegend	338819
Mouse Anti-Human CD184 PE	BD	557145

Table S4: **285 differentially expressed genes between UCB-derived CD34+ treated with DEN and UCB-derived CD34+ untreated (CTRL)**

Genes up-regulated in DEN treatment sorted according to fold change

Gene_Symbol	log2 FoldChange	P-value	Gene_Description
AGTR2	1.0744	0.014	angiotensin II receptor type 2
STOX1	0.9452	0.011	storkhead box 1
FAM218A	0.9066	0.001	family with sequence similarity 218 member A
SLC3A1	0.8987	0.016	solute carrier family 3 member 1
LINC00935	0.8965	0.018	None
PROX1	0.8960	0.011	prospero homeobox 1
RFPL4A	0.8853	0.011	ret finger protein like 4A
DAZ3	0.8603	0.026	deleted in azoospermia 3
PLET1	0.8488	0.013	placenta expressed transcript 1
LRRD1	0.8466	0.029	leucine rich repeats and death domain containing 1
CPB2	0.8064	0.002	carboxypeptidase B2
AK5	0.7573	0.003	adenylate kinase 5
GPC3	0.7504	0.020	glypican 3
GLT8D2	0.7496	0.004	glycosyltransferase 8 domain containing 2
PCDHB13	0.7346	0.050	protocadherin beta 13
C3orf20	0.7247	0.002	chromosome 3 open reading frame 20
SSMEM1	0.7217	0.032	serine rich single-pass membrane protein 1
CCDC89	0.7215	0.002	coiled-coil domain containing 89
PRKACG	0.7140	0.022	protein kinase cAMP-activated catalytic subunit gamma
DLEU1	0.7087	0.047	deleted in lymphocytic leukemia 1
SLC26A10	0.7077	0.013	solute carrier family 26 member 10
FAM205C	0.7077	0.012	family with sequence similarity 205 member C
CYP2B6	0.6969	0.005	cytochrome P450 family 2 subfamily B member 6
DEFB119	0.6962	0.003	defensin beta 119
SLC26A4	0.6862	0.015	solute carrier family 26 member 4
ETV1	0.6792	0.012	ETS variant transcription factor 1
SLC35F1	0.6789	0.011	solute carrier family 35 member F1
TNFRSF11A	0.6755	0.033	TNF receptor superfamily member 11a
STAB2	0.6741	0.011	stabilin 2
OR2B3	0.6733	0.026	olfactory receptor family 2 subfamily B member 3
CEP112	0.6680	0.026	centrosomal protein 112
TLL2	0.6663	0.007	tolloid like 2
PAGE5	0.6615	0.018	PAGE family member 5
SFTPA1	0.6613	0.005	surfactant protein A1
CHRNA9	0.6591	0.018	cholinergic receptor nicotinic alpha 9 subunit
HTR4	0.6480	0.008	5-hydroxytryptamine receptor 4
TFPI2	0.6464	0.018	tissue factor pathway inhibitor 2
SOX6	0.6461	0.022	SRY-box transcription factor 6
PRB1	0.6457	0.028	proline rich protein BstNI subfamily 1
SPRR2E	0.6395	0.012	small proline rich protein 2E
VSIG8	0.6382	0.026	V-set and immunoglobulin domain containing 8

FAM26E	0.6363	0.010	None
ADGRD1	0.6355	0.012	adhesion G protein-coupled receptor D1
GJB6	0.6308	0.005	gap junction protein beta 6
OR52I2	0.6291	0.028	olfactory receptor family 52 subfamily I member 2
DCDC2B	0.6283	0.004	doublecortin domain containing 2B
ALG1L	0.6259	0.017	ALG1 chitobiosyldiphosphodolichol beta-mannosyltransferase like
RTN4R	0.6239	0.027	reticulon 4 receptor
ACTRT1	0.6221	0.043	actin related protein T1
C12orf50	0.6207	0.012	chromosome 12 open reading frame 50
PYGO1	0.6170	0.009	pygopus family PHD finger 1
ITGB5	0.6139	0.035	integrin subunit beta 5
OR4K14	0.6111	0.014	olfactory receptor family 4 subfamily K member 14
NPY5R	0.6104	0.007	neuropeptide Y receptor Y5
APOBEC4	0.6095	0.004	apolipoprotein B mRNA editing enzyme catalytic polypeptide like 4
GABRG1	0.6082	0.024	gamma-aminobutyric acid type A receptor subunit gamma1
MSH4	0.6055	0.044	mutS homolog 4
CT45A5	0.6026	0.030	cancer/testis antigen family 45 member A5
ADTRP	0.5987	0.022	androgen dependent TFPI regulating protein
FRAT1	0.5985	0.018	FRAT regulator of WNT signaling pathway 1
YPEL2	0.5940	0.048	yippee like 2
CATSPER1	0.5904	0.022	cation channel sperm associated 1
GCOM1	0.5901	0.008	GCOM1, MYZAP-POLR2M combined locus
CACNA1B	0.5892	0.013	calcium voltage-gated channel subunit alpha1 B
SLC9B1	0.5818	0.014	solute carrier family 9 member B1
ZFHX3	0.5807	0.025	zinc finger homeobox 3
DEFB114	0.5774	0.013	defensin beta 114
OR8H1	0.5723	0.008	olfactory receptor family 8 subfamily H member 1
CA8	0.5691	0.010	carbonic anhydrase 8
LRRC71	0.5687	0.008	leucine rich repeat containing 71
TSHB	0.5645	0.018	thyroid stimulating hormone subunit beta
ADSSL1	0.5644	0.017	None
TEDDM1	0.5643	0.018	transmembrane epididymal protein 1
IGSF11	0.5597	0.019	immunoglobulin superfamily member 11
ELFN1	0.5555	0.007	extracellular leucine rich repeat and fibronectin type III domain containing 1
C9	0.5550	0.006	complement C9
FABP1	0.5547	0.023	fatty acid binding protein 1
FAM47E	0.5544	0.034	family with sequence similarity 47 member E
HOXC12	0.5521	0.018	homeobox C12
CDH18	0.5502	0.009	cadherin 18
OR5M8	0.5488	0.031	olfactory receptor family 5 subfamily M member 8
SRPX2	0.5479	0.029	sushi repeat containing protein X-linked 2
LMOD1	0.5476	0.011	leiomodin 1
FSCB	0.5471	0.027	fibrous sheath CABYR binding protein
WDR63	0.5451	0.008	None

SAMD15	0.5451	0.022	sterile alpha motif domain containing 15
ALKBH7	0.5446	0.014	alkB homolog 7
KU-MEL-3	0.5436	0.027	uncharacterized LOC497048
GPR142	0.5434	0.040	G protein-coupled receptor 142
OR56A3	0.5431	0.008	olfactory receptor family 56 subfamily A member 3
SBSN	0.5426	0.028	suprabasin
SPO11	0.5405	0.029	SPO11 initiator of meiotic double stranded breaks
ZMYND12	0.5397	0.011	zinc finger MYND-type containing 12
OR10Z1	0.5393	0.026	olfactory receptor family 10 subfamily Z member 1
DHDH	0.5390	0.046	dihydrodiol dehydrogenase
ZCCHC18	0.5353	0.012	zinc finger CCHC-type containing 18
SKIL	0.5324	0.013	SKI like proto-oncogene
FBXO47	0.5319	0.029	F-box protein 47
PMFBP1	0.5292	0.013	polyamine modulated factor 1 binding protein 1
CD164L2	0.5277	0.049	CD164 molecule like 2
ITIH2	0.5266	0.009	inter-alpha-trypsin inhibitor heavy chain 2
PKP3	0.5265	0.015	plakophilin 3
SPINK14	0.5262	0.030	serine peptidase inhibitor Kazal type 14
NUP210L	0.5257	0.042	nucleoporin 210 like
TMPRSS11E	0.5237	0.007	transmembrane serine protease 11E
CCDC34	0.5232	0.006	coiled-coil domain containing 34
RSPH1	0.5224	0.035	radial spoke head component 1
EQTN	0.5217	0.008	equatorin
RAB11A	0.5210	0.007	RAB11A, member RAS oncogene family
TUSC3	0.5143	0.018	tumor suppressor candidate 3
CD226	0.5098	0.017	CD226 molecule
OLAH	0.5071	0.037	oleoyl-ACP hydrolase
ADCYAP1R1	0.5056	0.009	ADCYAP receptor type I
MAPK4	0.5036	0.032	mitogen-activated protein kinase 4
PDCL2	0.5022	0.020	phosducin like 2
IL20RA	0.5015	0.011	interleukin 20 receptor subunit alpha
OR2A14	0.5008	0.008	olfactory receptor family 2 subfamily A member 14
OR2T35	0.5002	0.029	olfactory receptor family 2 subfamily T member 35
DNAH12	0.4997	0.014	dynein axonemal heavy chain 12
OR10H2	0.4989	0.036	olfactory receptor family 10 subfamily H member 2
IL15	0.4985	0.044	interleukin 15
STARD6	0.4944	0.042	StAR related lipid transfer domain containing 6
CNIH2	0.4934	0.017	cornichon family AMPA receptor auxiliary protein 2
ANKRD44	0.4933	0.013	ankyrin repeat domain 44
MEI4	0.4929	0.047	meiotic double-stranded break formation protein 4
SRRM4	0.4917	0.014	serine/arginine repetitive matrix 4

Genes down-regulated in DEN treatment sorted according to fold change

Gene_Symbol	log2 FoldChange	P-value	Gene_Description
CXCL10	-1.4099	0.010	C-X-C motif chemokine ligand 10
CCDC36	-1.3728	0.000	None
CD86	-1.3287	0.000	CD86 molecule
ZBBX	-1.1794	0.001	zinc finger B-box domain containing
MAGEB18	-1.1481	0.036	MAGE family member B18
HRH4	-1.0544	0.013	histamine receptor H4
UGT2B10	-1.0352	0.003	UDP glucuronosyltransferase family 2 member B10
GJA8	-0.9781	0.005	gap junction protein alpha 8
TNFRSF9	-0.9507	0.046	TNF receptor superfamily member 9
SLC25A21	-0.9047	0.006	solute carrier family 25 member 21
RBM46	-0.9006	0.037	RNA binding motif protein 46
C8orf4	-0.8824	0.001	None
GLYATL2	-0.8669	0.042	glycine-N-acyltransferase like 2
UGT2B17	-0.8463	0.005	UDP glucuronosyltransferase family 2 member B17
C10orf62	-0.8436	0.001	chromosome 10 open reading frame 62
INS	-0.8300	0.001	insulin
UGT3A1	-0.8260	0.001	UDP glycosyltransferase family 3 member A1
USP13	-0.8195	0.011	ubiquitin specific peptidase 13
KIZ	-0.8114	0.002	kizuna centrosomal protein
SH2D1B	-0.8031	0.019	SH2 domain containing 1B
OR1L8	-0.7990	0.045	olfactory receptor family 1 subfamily L member 8
OR10A5	-0.7967	0.001	olfactory receptor family 10 subfamily A member 5
PIFO	-0.7914	0.003	primary cilia formation
GPR34	-0.7913	0.011	G protein-coupled receptor 34
CHI3L2	-0.7856	0.014	chitinase 3 like 2
OR5D14	-0.7852	0.008	olfactory receptor family 5 subfamily D member 14
C2orf15	-0.7848	0.001	chromosome 2 open reading frame 15
CTSV	-0.7789	0.010	cathepsin V
FCRL2	-0.7787	0.036	Fc receptor like 2
OR2T10	-0.7764	0.001	olfactory receptor family 2 subfamily T member 10
TMEM14EP	-0.7732	0.016	transmembrane protein 14E, pseudogene
IZUMO1R	-0.7616	0.003	IZUMO1 receptor, JUNO
TNNC1	-0.7610	0.004	troponin C1, slow skeletal and cardiac type
HIPK4	-0.7522	0.008	homeodomain interacting protein kinase 4
EDN2	-0.7519	0.007	endothelin 2
TRDN	-0.7452	0.003	triadin
SUSD4	-0.7393	0.001	sushi domain containing 4
SLC22A10	-0.7306	0.005	solute carrier family 22 member 10
HTN3	-0.7300	0.016	histatin 3
ATP13A4	-0.7262	0.009	ATPase 13A4
UGT8	-0.7249	0.004	UDP glycosyltransferase 8
OR52E1	-0.7236	0.005	olfactory receptor family 52 subfamily E member 1

OR2A25	-0.7202	0.002	olfactory receptor family 2 subfamily A member 25
RGS13	-0.7166	0.028	regulator of G protein signaling 13
CRYGB	-0.7151	0.013	crystallin gamma B
KLRF1	-0.7114	0.004	killer cell lectin like receptor F1
C4orf22	-0.7036	0.030	None
UNC5C	-0.6944	0.012	unc-5 netrin receptor C
PKD1L3	-0.6914	0.004	polycystin 1 like 3, transient receptor potential channel interacting
CSMD3	-0.6907	0.006	CUB and Sushi multiple domains 3
FEZ1	-0.6880	0.035	fasciculation and elongation protein zeta 1
CAGE1	-0.6873	0.003	cancer antigen 1
C16orf46	-0.6845	0.015	chromosome 16 open reading frame 46
TRPC3	-0.6831	0.023	transient receptor potential cation channel subfamily C member 3
NR1H4	-0.6772	0.031	nuclear receptor subfamily 1 group H member 4
LAMA1	-0.6768	0.006	laminin subunit alpha 1
IL22	-0.6759	0.003	interleukin 22
MRC1	-0.6710	0.039	mannose receptor C-type 1
BMP8A	-0.6635	0.036	bone morphogenetic protein 8a
OR5AN1	-0.6614	0.010	olfactory receptor family 5 subfamily AN member 1
INSL4	-0.6573	0.038	insulin like 4
ADH1A	-0.6535	0.033	alcohol dehydrogenase 1A (class I), alpha polypeptide
BOLL	-0.6522	0.019	boule homolog, RNA binding protein
LMNTD1	-0.6504	0.023	lamin tail domain containing 1
PTCHD3	-0.6481	0.029	patched domain containing 3
HNF1B	-0.6459	0.012	HNF1 homeobox B
A3GALT2	-0.6418	0.033	alpha 1,3-galactosyltransferase 2
MPZ	-0.6381	0.049	myelin protein zero
AC008753.6	-0.6373	0.008	None
AADAC	-0.6365	0.008	arylacetamide deacetylase
PRICKLE1	-0.6360	0.032	prickle planar cell polarity protein 1
CSAD	-0.6325	0.009	cysteine sulfinic acid decarboxylase
ACSBG2	-0.6293	0.003	acyl-CoA synthetase bubblegum family member 2
THPO	-0.6290	0.005	thrombopoietin
GJB4	-0.6286	0.004	gap junction protein beta 4
APOA2	-0.6264	0.008	apolipoprotein A2
TM4SF1	-0.6252	0.008	transmembrane 4 L six family member 1
SLC9A2	-0.6249	0.004	solute carrier family 9 member A2
PTPRK	-0.6209	0.045	protein tyrosine phosphatase receptor type K
CEMIP	-0.6205	0.005	cell migration inducing hyaluronidase 1
CACNA2D1	-0.6189	0.008	calcium voltage-gated channel auxiliary subunit alpha2delta 1
LRP2	-0.6163	0.045	LDL receptor related protein 2
TDRD1	-0.6163	0.036	tudor domain containing 1
OR5B12	-0.6137	0.021	olfactory receptor family 5 subfamily B member 12

OR1B1	-0.6127	0.038	olfactory receptor family 1 subfamily B member 1
GALNTL6	-0.6091	0.008	polypeptide N-acetylgalactosaminyltransferase like 6
ERBB3	-0.6074	0.008	erb-b2 receptor tyrosine kinase 3
UBAP1L	-0.6071	0.021	ubiquitin associated protein 1 like
HOXD4	-0.6024	0.024	homeobox D4
DZANK1	-0.5970	0.029	double zinc ribbon and ankyrin repeat domains 1
LOC653653	-0.5966	0.040	adaptor-related protein complex 1, sigma 2 subunit (AP1S2) pseudogene
SHC3	-0.5875	0.031	SHC adaptor protein 3
SLC16A6	-0.5869	0.038	solute carrier family 16 member 6
KRTAP4-2	-0.5867	0.004	keratin associated protein 4-2
DPPA5	-0.5841	0.004	developmental pluripotency associated 5
LINC00272	-0.5773	0.016	long intergenic non-protein coding RNA 272
IL36B	-0.5762	0.022	interleukin 36 beta
DRAXIN	-0.5755	0.014	dorsal inhibitory axon guidance protein
KRTAP6-1	-0.5748	0.024	keratin associated protein 6-1
TMEM176B	-0.5743	0.011	transmembrane protein 176B
ALOX15B	-0.5729	0.047	arachidonate 15-lipoxygenase type B
EVX1	-0.5723	0.035	even-skipped homeobox 1
RORA	-0.5691	0.007	RAR related orphan receptor A
OTOA	-0.5679	0.049	otoancorin
VPS16	-0.5673	0.019	VPS16 core subunit of CORVET and HOPS complexes
CC2D2B	-0.5641	0.049	coiled-coil and C2 domain containing 2B
SERPINB10	-0.5627	0.025	serpin family B member 10
NALCN	-0.5607	0.015	sodium leak channel, non-selective
KRTAP19-3	-0.5604	0.005	keratin associated protein 19-3
C1orf137	-0.5581	0.025	None
KCNH5	-0.5568	0.018	potassium voltage-gated channel subfamily H member 5
RAB40A	-0.5561	0.010	RAB40A, member RAS oncogene family
ZNF781	-0.5548	0.021	zinc finger protein 781
CLRN1	-0.5524	0.021	clarin 1
FMR1NB	-0.5505	0.005	FMR1 neighbor
SLC6A19	-0.5496	0.030	solute carrier family 6 member 19
IGSF5	-0.5490	0.016	immunoglobulin superfamily member 5
LINC01465	-0.5488	0.027	long intergenic non-protein coding RNA 1465
RNF222	-0.5478	0.007	ring finger protein 222
MOXD1	-0.5477	0.007	monooxygenase DBH like 1
PIPOX	-0.5475	0.008	pipecolic acid and sarcosine oxidase
FOXD4L3	-0.5462	0.020	forkhead box D4 like 3
ADAMTS12	-0.5413	0.008	ADAM metallopeptidase with thrombospondin type 1 motif 12
SYNC	-0.5379	0.044	syncoilin, intermediate filament protein
COL4A6	-0.5365	0.022	collagen type IV alpha 6 chain
PCDH20	-0.5340	0.037	protocadherin 20
TG	-0.5332	0.009	thyroglobulin
LOC730183	-0.5323	0.013	novel transcript, antisense to SRCAP
NPPA	-0.5313	0.032	natriuretic peptide A

C10orf111	-0.5304	0.007	None
C8orf89	-0.5294	0.032	chromosome 8 open reading frame 89
RPGRIP1	-0.5288	0.015	RPGR interacting protein 1
KRT39	-0.5246	0.020	keratin 39
GOLGA6B	-0.5233	0.014	golgin A6 family member B
MUC17	-0.5212	0.019	mucin 17, cell surface associated
CCR1	-0.5201	0.017	C-C motif chemokine receptor 1
RNASE7	-0.5198	0.041	ribonuclease A family member 7
FPR3	-0.5187	0.019	formyl peptide receptor 3
KISS1R	-0.5174	0.021	KISS1 receptor
TMPRSS7	-0.5169	0.010	transmembrane serine protease 7
NEUROD1	-0.5168	0.025	neuronal differentiation 1
BTBD18	-0.5105	0.007	BTB domain containing 18
OR2L8	-0.5100	0.022	olfactory receptor family 2 subfamily L member 8
DEFB116	-0.5077	0.036	defensin beta 116
WNT2	-0.5055	0.012	Wnt family member 2
KLK9	-0.5051	0.031	kallikrein related peptidase 9
KRTAP20-1	-0.5049	0.019	keratin associated protein 20-1
FAM183A	-0.5028	0.014	family with sequence similarity 183 member A
RBM44	-0.5017	0.021	RNA binding motif protein 44
OR10K2	-0.5007	0.009	olfactory receptor family 10 subfamily K member 2
ISPD	-0.4994	0.021	None
PCDH8	-0.4990	0.027	protocadherin 8
IFNG	-0.4988	0.042	interferon gamma
HTR6	-0.4981	0.033	5-hydroxytryptamine receptor 6
CACNA1A	-0.4969	0.019	calcium voltage-gated channel subunit alpha1 A
HOXC8	-0.4964	0.034	homeobox C8
PP14571	-0.4949	0.029	None
ZNF133	-0.4947	0.022	zinc finger protein 133
GIMD1	-0.4945	0.014	GIMAP family P-loop NTPase domain containing 1

Table S5. Functional Gene Ontology (GO) categories.

Processes	pValue	FDR	In Data	Network Objects from Active Data
**cell-cell signaling**	1.153E-09	6.609E-06	50	TSH-beta, PSMA4, SPAPI, Galpha(i)-specific amine GPCRs, NeuroD1, Connexin 30, HNF1-beta, IL-15, N-type Ca(II) channel alpha1B, HTR4, Connexin 50, CCR1, PYGO1, AGTR2, Insulin, HNF1, PKA-cat (cAMP-dependent), PCDHB6, FRAT1, Olfactory receptor, Galpha(q)-specific peptide GPCRs, CNIH2, INSL4, SHC3, nAChR alpha-9, OR10H2, NPY5R, Myelin P0 protein, Galpha(i)-specific peptide GPCRs, P/Q-type calcium channel alpha-1A subunit, WNT2, HDL proteins, C1orf187, Galpha(s)-specific amine GPCRs, LRP2 (Megalin), Connexin 30.3, nAChR alpha, CACNA2D1, RANK(TNFRSF11A), GABRG1, IP10, Histamine H4 receptor, HTR6, Prickle-1, Serotonin receptor, PCDH8, WNT, PCDHB13, CACNA2D, Galpha(q)-specific PACAP GPCR
**G protein-coupled receptor signaling pathway**	3.138E-08	2.436E-05	8	Galpha(i)-specific amine GPCRs, HTR4, Olfactory receptor, OR10H2, Galpha(s)-specific amine GPCRs, Histamine H4 receptor, HTR6, Serotonin receptor
**regulation of cytosolic calcium ion concentration**	4.415E-08	2.436E-05	24	Galpha(i)-specific amine GPCRs, N-type Ca(II) channel alpha1B, CCR1, PKA-cat (cAMP-dependent), Endothelin-2, FPRL2, Galpha(q)-specific peptide GPCRs, Triadin, nAChR alpha-9, TMEM2L, Galpha(i)-specific peptide GPCRs, P/Q-type calcium channel alpha-1A subunit, TRPC3, Galpha(s)-specific amine GPCRs, nAChR alpha, PACAP receptor 1, CACNA2D1, IP10, Histamine H4 receptor, Serotonin receptor, TRPC, WNT, CACNA2D, Galpha(q)-specific PACAP GPCR
**positive regulation of cytosolic calcium ion concentration**	5.40E-08	2.44E-05	22	Galpha(i)-specific amine GPCRs, N-type Ca(II) channel alpha1B, CCR1, Endothelin-2, FPRL2, Galpha(q)-specific peptide GPCRs, Triadin, nAChR alpha-9, TMEM2L, Galpha(i)-specific peptide GPCRs, P/Q-type calcium channel alpha-1A subunit, TRPC3, Galpha(s)-specific amine GPCRs, nAChR alpha, PACAP receptor 1, CACNA2D1, IP10, Histamine H4 receptor, Serotonin receptor, TRPC, CACNA2D, Galpha(q)-specific PACAP GPCR
**C-X-C chemokine receptor CXCR4 signaling pathway**	4.03E-04	5.81E-03	2	Galpha(q)-specific peptide GPCRs, Galpha(i)-specific peptide GPCRs
**CXCL12-activated CXCR4 signaling pathway**	4.03E-04	5.81E-03	2	Galpha(q)-specific peptide GPCRs, Galpha(i)-specific peptide GPCRs

**positive regulation of cytokine biosynthetic process**	5.29E-04	6.91E-03	5	Galpha(q)-specific peptide GPCRs, CD86, IFN-gamma, Galpha(i)-specific peptide GPCRs, WNT
**chemokine-mediated signaling pathway**	4.90E-03	3.08E-02	5	CCR1, Thrombopoietin, Galpha(q)-specific peptide GPCRs, Galpha(i)-specific peptide GPCRs, IP10
**regulation of G protein-coupled receptor signaling pathway**	4.48E-03	2.92E-02	9	Galpha(i)-specific amine GPCRs, RGS18, Galpha(q)-specific peptide GPCRs, Tissue kallikreins, Galpha(i)-specific peptide GPCRs, Galpha(s)-specific amine GPCRs, RGS13, Serotonin receptor, WNT
**cell surface receptor signaling pathway**	4.30E-03	2.82E-02	57	ANP, RPTPkappa, ROR-alpha, PSMA4, SPAPI, Galpha(i)-specific amine GPCRs, HNF1-beta, IL-15, CCR1, PYGO1, BMP8A, AGTR2, Thrombopoietin, Insulin, CD137(TNFRSF9), PKA-cat gamma, Mucin 17, HNF1, PKA-cat (cAMP-dependent), Endothelin-2, FRAT1, FPRL2, EAT-2, UNC5C, KLRF1, Galpha(q)-specific peptide GPCRs, Zcchc18, LAMA1, CD86, SHC3, nAChR alpha-9, GPR133, IFN-gamma, IL20RA, GPR34, Galpha(i)-specific peptide GPCRs, WNT2, ITGB5, HDL proteins, Sno-N, C1orf187, Galpha(s)-specific amine GPCRs, COL4A6, IL-1F8, nAChR alpha, PACAP receptor 1, RANK(TNFRSF11A), IP10, RTN4R, Prickle-1, FCRLB, IL-22, WNT, FXR, Galpha(q)-specific PACAP GPCR, Collagen IV, ErbB3
**negative regulation of cell-matrix adhesion**	1.23E-01	2.24E-02	2	HDL proteins, C11orf34
**extracellular structure organization**	1.48E-01	2.54E-02	9	TLL2, LAMA1, Tissue kallikreins, ITGB5, COL4A6, Cathepsin V, WNT, ADAM-TS12, Collagen IV
**regulation of cell-matrix adhesion**	1.48E-01	2.54E-02	4	Galpha(i)-specific peptide GPCRs, HDL proteins, WNT, C11orf34
**regulation of stem cell population maintenance**	1.48E-01	2.54E-02	9	TLL2, LAMA1, Tissue kallikreins, ITGB5, COL4A6, Cathepsin V, WNT, ADAM-TS12, Collagen IV

Supplementary Figures

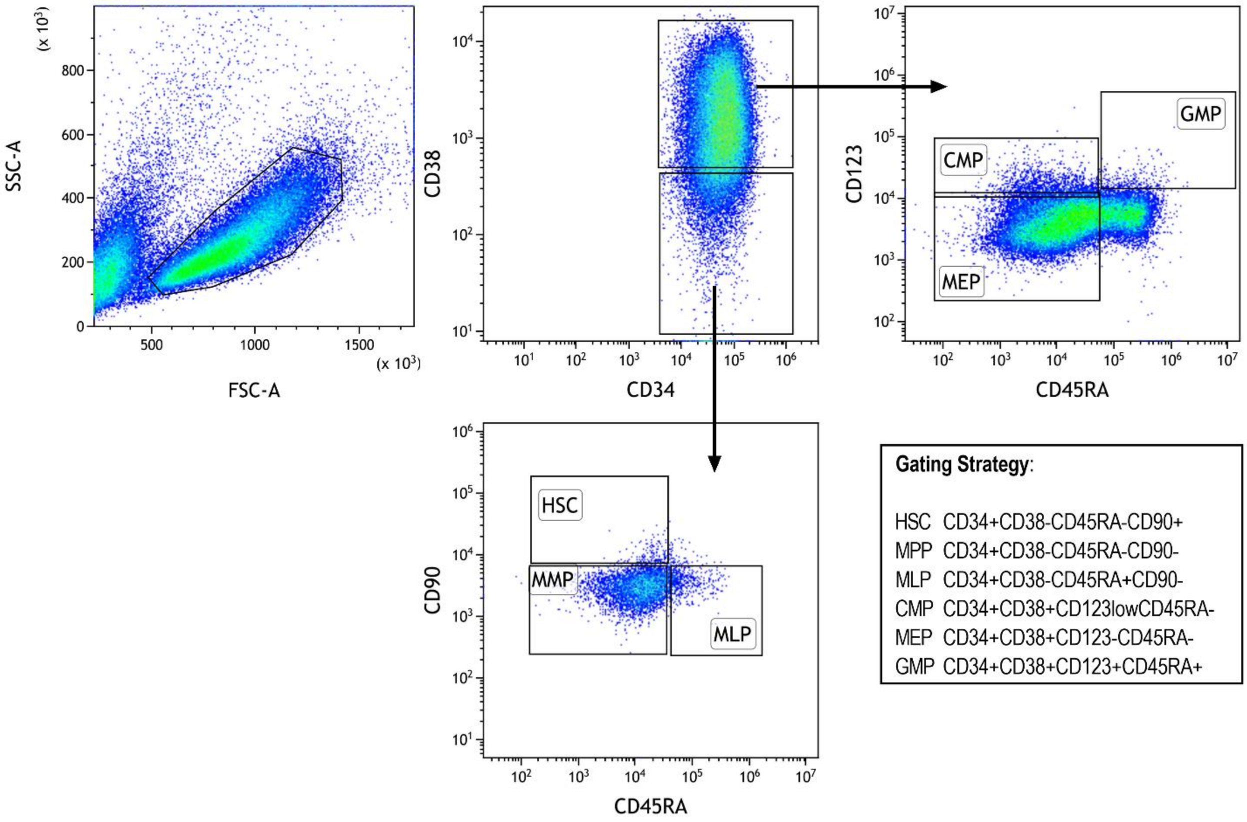

Supplementary figure 1. Gating strategy for identification of hematopoietic stem and progenitor cell population

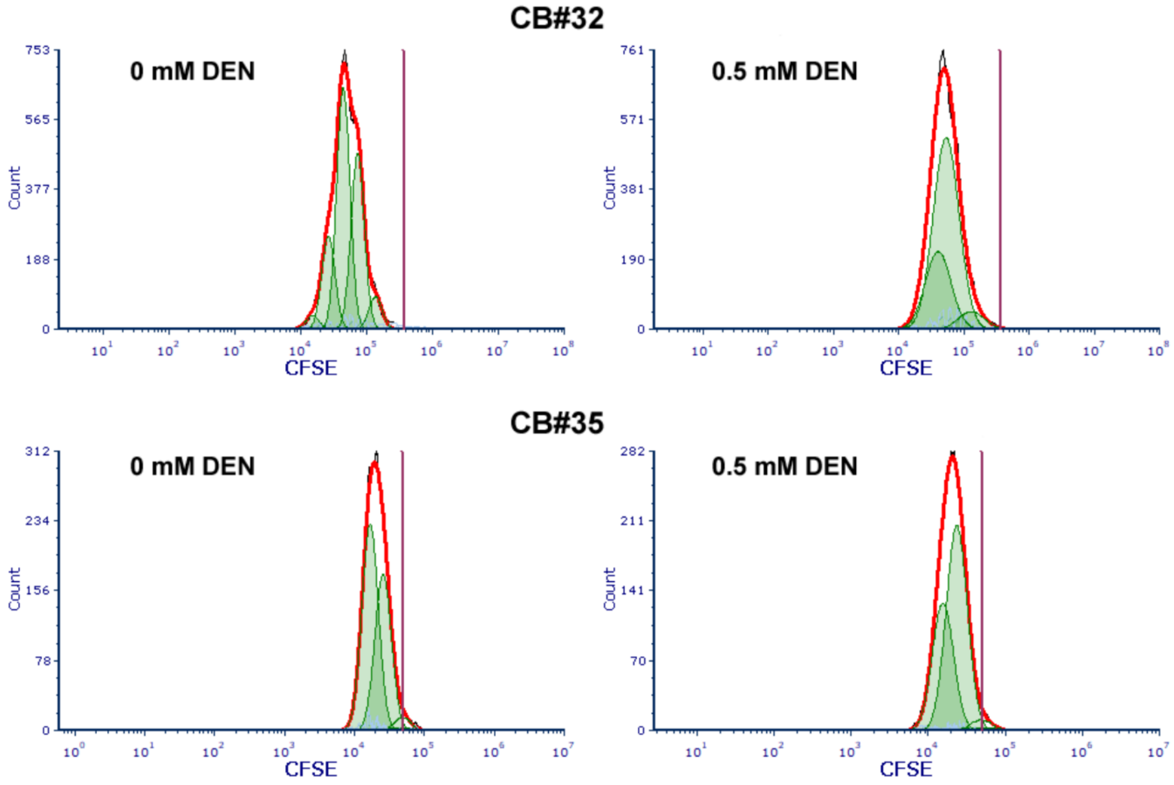

Supplementary figure 2. Representative proliferation analysis determined by CFSE staining of two samples of UCB-derived CD34+ cells cultured for six days in the presence of SCF, IL-3 and GM-CSF with or without 0.5mM DEN.

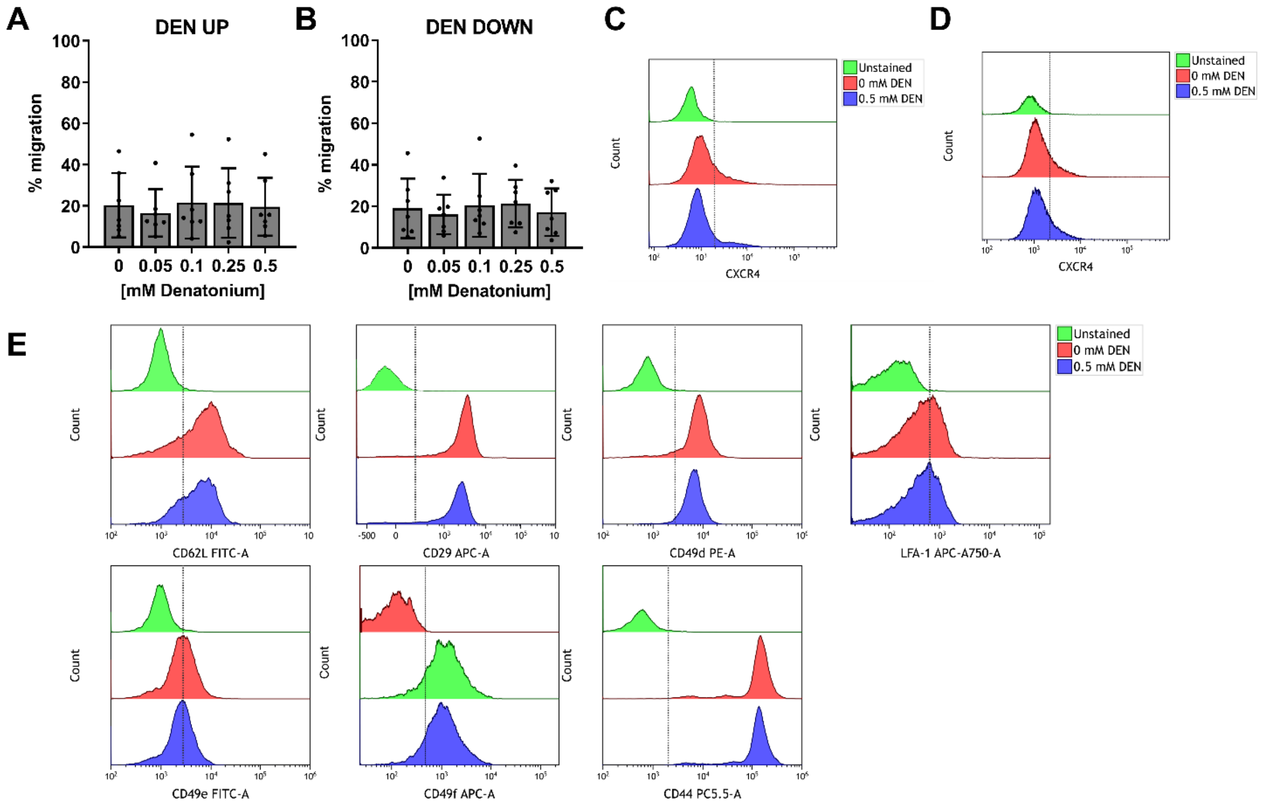

Supplementary figure 3 Spontaneous migration of UCB-derived CD34+ cells (A) in the presence of increasing doses of DEN in the upper chamber of the transwell system (n=7) or (B) towards a gradient of DEN in the lower chamber of transwell system (n=7). Results are shown as mean ± SD (C) Representative FACS analysis of CXCR4 expression after 24h exposure to 0.5mM DEN. (D) Representative FACS analysis of CXCR4 expression 4h post a 24h pre-treatment with 0.5 mM DEN. (E) Representative FACS analysis of the adhesion molecules expression in UCB-derived CD34+ cells treated with 0.5 mM DEN. Data represent mean ± SD.
